# Comparison Between the Effects of Transfer Energy Capacitive and Resistive Therapy and Therapeutic Ultrasound on Hamstring Muscle Shortness in Male Athletes: A Single-Blind Randomized Controlled Trial

**DOI:** 10.31661/gmj.v12i.2981

**Published:** 2023-08-19

**Authors:** Haniyeh Choobsaz, Nastaran Ghotbi, Pooria Mohamadi

**Affiliations:** ^1^ Department of Physiotherapy, School of Rehabilitation, Tehran University of Medical Sciences, Tehran, Iran

**Keywords:** Hamstring Muscle, Physical Therapy Modalities, Static Stretching, Radiofrequency Therapy, Diathermy

## Abstract

Background: Transfer energy capacitive and resistive (TECAR) therapy (TT) is a newly developed deep heating therapy that can generate heat within tissues through high-frequency wave stimulation. Compared to conventional physiotherapy methods, the application of TT especially in sports rehabilitation is becoming more popular. This study aimed to investigate the comparative effect of TT and therapeutic ultrasound (US) on hamstring muscle shortness. Additionally, the effects of TT with static stretching (SS) were compared with US combined with SS.Materials and Methods: Totally, 39 male athletes with hamstring shortness were randomly assigned into three groups: A, B, and C. Group A received 15 minutes of TT plus SS, while Group B received 15 minutes of US with SS, and Group C only performed SS. Hamstring flexibility was measured by active knee extension (AKE), passive knee extension (PKE), and the sit and Reach (SR) tests before the intervention, and following the first, and third treatment sessions.Results: The range of motion of the AKE and PKE, and displacement range in the SR test improved significantly after the first and third sessions in all three groups (P0.0001). The improvement of the three flexibility indices in the TT group was greater than in the other two groups.Conclusion: The present study showed that TT could increase the flexibility of hamstring muscles more than US therapy. However, TT in combination with SS had a similar effect to SS alone.

## Introduction

**Figure-1:**
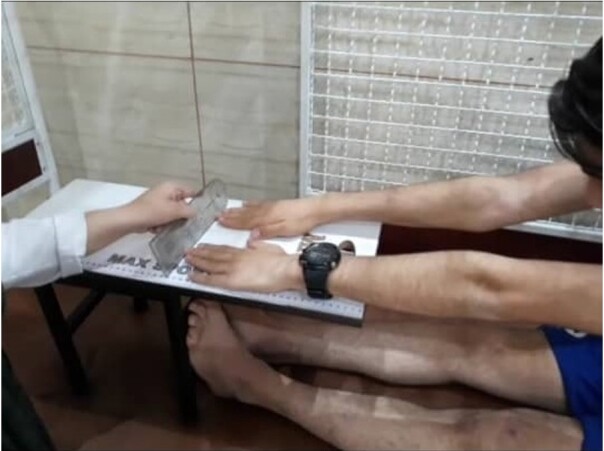
Figure-1. Sit & Reach test to evaluate hamstring muscle flexibility

**Figure-2:**
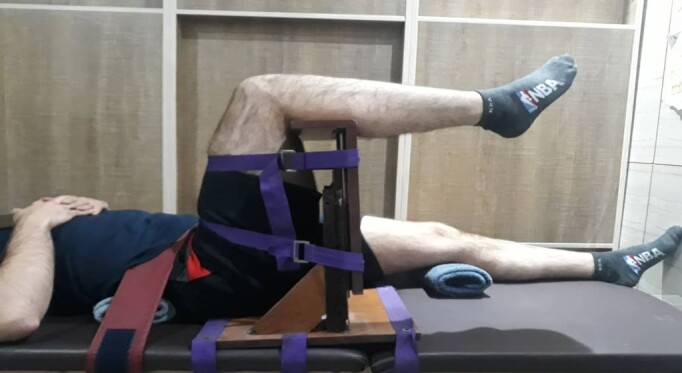
Figure-2. Athlete's position for the measurement of Active Knee Extension

Muscle flexibility is an important component of normal biomechanical functioning in athletes [1]. Insufficient muscle flexibility may cause musculoskeletal injuries [2]. According to studies, the reduced flexibility of hamstrings influences athletic performance and is a risk factor for developing hamstrings strain [3]. Therefore, the maintenance of the flexibility of hamstrings in athletes is a major issue in physiotherapy.

Various methods are used to improve the muscle flexibility of athletes, including stretching and thermotherapy [4]. Static stretching (SS), due to its low risk of inducing injury, is one of the most effective techniques to improve muscle flexibility [5][6]. Thermotherapy is another means of enhancing muscle flexibility, increasing tissue temperature and blood flow and reducing muscle activity [7][8]. Deep heating therapy can be applied through various modalities including ultrasound (US) [9], short-wave diathermy [10], and microwaves [11] which have been shown to have different clinical effectiveness.

It is believed that deep heating agents can increase the extensibility of collagen fibers by increasing intramuscular temperature [12]. In this way, more significant muscle elongation can be achieved even at lower stretch forces [13]. Low-frequency continuous therapeutic US can penetrate deep tissue layers containing thick muscles to improve muscle flexibility [14][15].

Recently, Transfer Energy Capacitive and Resistive (TECAR) therapy (TT) has been used for clinical purposes as almost a unique physiotherapy modality [16][17][18]. It consists of an electrical current, which induces deep endogenous heating by a 448 kHz capacitive/resistive monopolar radiofrequency [19][20], and can increase the extensibility of soft tissues and muscle flexibility [21][22]. Ribeiro et al. assessed the effectiveness of TT in musculoskeletal disorders. They concluded that this is one of the best modalities of physiotherapy whether used alone or integrated into conventional rehabilitation, with both short-term and long-term benefits. [21]. Recently, TT has been introduced to improve hamstring muscle flexibility [23][24]. However, it is unclear whether its effect on muscle elongation is more than the other mentioned methods (i.e., therapeutic US or SS). In our research, no study was found comparing the effects of TT with the therapeutic US on hamstring muscle shortness. Thus, this study aimed to compare the effects of TT and US on the flexibility of hamstrings in healthy athletes with short hamstrings. Furthermore, the effects of US with SS, and TT with SS were compared separately.

## Materials and Methods

**Figure-3:**
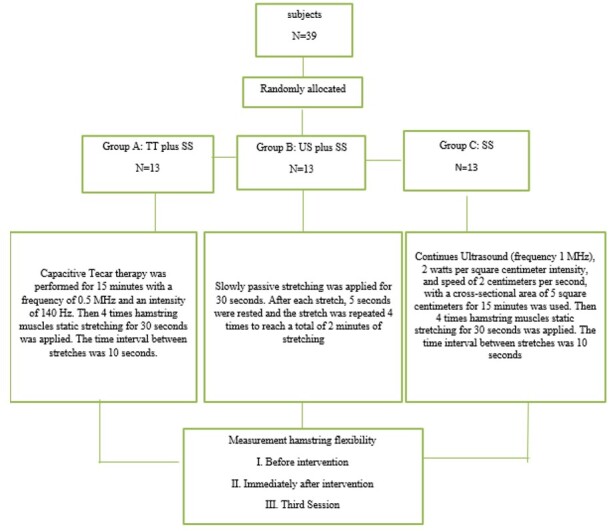
Figure-3. Summary of groups intervention

**Table:** Table1. Demographic Data of Athletes in Three Groups

Variable	Group A (n=13) Mean (SD)	Group B (n=13) Mean (SD)	Group C (n=13) Mean (SD)	P-Value
Age (years)	22.9(2.431)	24.38(2.364)	24.54(3.017)	0.23
Weight (kg)	69.62(4.053)	68.58(7.457)	72.38(4.92)	0.15
Height (cm)	179.54(4.648)	179.15(4.259)	181.54(2.847)	0.27
BMI (kg/m^2^)	21.592(0.982)	21.538(1.077)	21.938(0.9134)	0.54

^*^BMI: Body Mass Index

**Table:** Table2. Main Group Interaction and Evaluation Time for Hamstring Muscle Flexibility

Variables	Source	Type III Sum of Squares	D.F	Mean Square	F	Sig.	Partial Eta Squared
SRT	Time	66.462	1.190	55.856	14.526	.000 ^*^	.287
	Time * Group	38.154	2.380	16.033	4.169	.017 ^*^	.188
	Group	14.923	2	7.462	.971	.388	.051
PKE	Time	238.308	1.633	145.936	86.995	.000 ^*^	.707
	Time * Group	74.410	3.266	22.784	13.582	.000 ^*^	.430
	Group	143.744	2	71.872	5.882	.006 ^*^	.246
AKE	Time	291.897	1.729	168.826	72.178	.000 ^*^	.667
	Time * Group	82.513	3.458	23.862	10.201	.000 ^*^	.362
	Group	222.205	2	111.103	9.088	.001 ^*^	.336

**SRT:** Sit and Reach Test; **PKE:** Passive Knee Extension; **AKE:** Active Knee Extension *P<0.05 was significant

Study Subjects

A total of 39 non-professional male athletes with hamstring muscle shortness participated in the study [2]. The knee extension range of motion (ROM) of 70 degrees or less in the passive knee extension (PKE) test of the dominant leg was considered hamstring shortness [24]. The exclusion criteria were that the athlete had a history of orthopedic or neurological disorders in the lower limbs within the last six months. In addition, subjects with contraindications to TECAR or US were excluded from the study. The procedure was explained to the participants before obtaining their informed consent. The study was approved by the University’s Ethics Committee (IR.TUMS.FNM.REC.1398.063 ; (IRCT20190920044826N1)). The athletes were randomly assigned into groups of A, B, and C by a simple randomization method (using dice-throwing). One physiotherapist applied the interventions, and another physiotherapist who was unaware of the type of intervention performed the measurements.

Study Assessment

The Sit and Reach (SR) test (Figure-1) as well as active knee extension (AKE) and PKE tests (Figure-2), were used to measure hamstring muscle flexibility [25][26] using a Flex Tester Box and an orthopedic goniometer (Maurice E. Mueller Foundation, Bern, Switzerland), respectively. All measurements were carried out three times before the intervention and after the first and third sessions.

Study Intervention

Interventions included TT, SS, and US therapy administered on a group basis. Participants in each group received treatment three times a week. All interventions were applied in the following manner:

Group A: TT plus SS

The subjects received TECAR therapy (Exon Medical company, TecaTen model IRAN-Class B) in a prone position for 15 minutes at a frequency of 0.5 (MHz). The active electrode (5 cm2) had a continuous circular motion over the posterior surface of the thigh, while the inactive electrode was placed stationary over the quadriceps muscles (170 mm 230 mm). Then, the hamstrings were stretched four times for 30 seconds each. The time interval between stretches was 10 seconds.

Group B: US plus SS

Continuous US (Nutek model Pro UT1041, China) was applied to the hamstrings for 15 minutes while the subjects were in a prone position. US therapy was administered with a power of 2 W/cm2, a frequency of 1 MHz, and a cross-sectional probe area of 5 cm2 with a circular motion speed of 2 cm per second. Subsequently, the hamstrings were stretched four times for 30 seconds each. The period between stretches was 10 seconds.

Group C: SS

Passive stretching was applied in a supine position. The hip was flexed to 90° and the knee was passively and slowly extended [27]. To fix the pelvis, the therapist pushed down against the opposite leg (to prevent posterior pelvic tilt and lumbar flexion). Each stretching technique was performed for 30 seconds and repeated four times. There was a 5-second pause between each stretching technique (Figure-3).

Statistical Analysis

The obtained data were analyzed using SPSS version 21(SPSS Inc; Chicago, IL, USA). The Kolmogorov-Smirnov test showed normal distribution for all variables (P>0.05). A one-way analysis of variance (ANOVA) was used to examine demographic differences between groups, as well as, determine the differences in flexibility indices (AKE & PKE ROM, and SR displacement) among the groups before treatment.

A two-way ANOVA was used to detect differences between groups and time points. A Tukey Honestly Significant Difference (HSD) post hoc analysis was performed to interpret the findings. Also, Cohen’s d was used to determine the effect size of the groups on hamstring flexibility indices. The level of significance for all tests was set at P<0.05.

## Results

**Table:** Table3. Indices of Hamstring Flexibility (Knee Extension ROM and the Sit and Reach Test) between the Three Treatment Groups

Variables	(I) GROUP	(J) GROUP	Mean Difference (I-J)	Std. Error	P Value	95% Confidence Interval	
						Lower Bound	Upper Bound
SRT	Group A	Group B	.23	.628	1.000	-1.35	1.81
	Group B	Group C	-.85	.628	.558	-2.42	.73
	Group C	Group A	.62	.628	1.000	-.96	2.19
PKE	Group A	Group B	2.26 ^*^	.792	.022*	.27	4.24
	Group B	Group C	-2.44 ^*^	.792	.012*	-4.42	-.45
	Group C	Group A	.18	.792	1.000	-1.81	2.17
AKE	Group A	Group B	2.95 ^*^	.792	.002*	.96	4.94
	Group B	Group C	-2.90 ^*^	.792	.002*	-4.89	-.91
	Group C	Group A	-.05	.792	1.000	-2.04	1.94

**SRT:** Sit and Reach Test; **PKE:** Passive Knee Extension; **AKE:** Active Knee Extension **Group A:** TECAR Therapy; **Group B:** Ultrasound therapy; **Group C:** Passive stretching *P<0.05 was significant

**Table:** Table4. Effect Size Values in Muscle Flexibility Indices

Test	Group A	Group B	Group C
PKE	1.60	1.12	1.44
AKE	3.31	0.65	2.60
SR	1.24	0.53	0.55

**SR:** Sit and Reach; **PKE:** Passive Knee Extension; **AKE:** Active Knee Extension

A total of 39 athletes participated in the study. The baseline measurements of the participants are presented in Table-1. One-way ANOVA test revealed no significant differences between groups in age, weight, height, and body mass index (BMI). Furthermore, no significant difference was found between the PKE and AKE ROM, and displacement range in the SR test before treatment among the groups (P>0.05). Two-way mixed model ANOVA revealed significant differences in AKE (P=0.001) and PKE (P=0.006) ROM among the three groups. However, the displacements in the SR test were not statistically significant among the groups (P=0.38). The effect of assessment time on hamstring muscle flexibility indices was significant in all three groups (P<0.05). The mean of all test variables after the third session was more than the first and the pre-treatment sessions (P<0.05). The effects of group interaction and assessment time were significant for all three indices (P<0.05) (Table-2). Tukey’s HSD post hoc test showed that in groups A and C, the mean ROM of the AKE and PKE was statistically greater than that of group B (P<0.05). However, there was no difference between groups A and C (P>0.05).

Displacement range in the SR test did not show a statistically significant difference among the three treatment groups (P<0.05, Table-3). Cohen’s d was used to determine the extent of the effect of the different interventions on hamstring flexibility indices in the three groups. Values between 0.2 and 0.5 were interpreted as weak, while values between 0.5 and 0.8, and above 0.80 were considered as medium and strong effect sizes, respectively (Cohen J. Statistical Power Analysis for the Behavioral Sciences; Brydges, 2019 #72). The results showed that the effect size of Group A on changes for the AKE and PKE ROM, and displacement range for the SR test was strong. Group B had a strong effect on ROM changes for AKE and PKE, while it was moderate for the SR test. Group C effect size on the AKE and PKE ROM was strong, but the range of displacement in the SR test showed a moderate effect (Table-4).

## Discussion

This study was primarily conducted to compare the effects of TT and US therapy on hamstring muscle flexibility in male athletes with hamstring muscle shortness. The results showed that TT could improve hamstring flexibility more than US therapy in all measures. One possible explanation may be attributed to the different nature of these modalities. TECAR is a radiofrequency wave that can increase the endogenous temperature in biological structures [18][22]. We used the capacitive energy transfer mode, which affects the tissues that contain more electrolytes, including muscles and soft tissues [28]. On the other hand, the US mechanical acoustic wave is also absorbed by the muscles as protein-rich tissues [29]. However, high-frequency diathermy waves like TECAR can affect a much wider area than the US [30]. In other words, although the cross-sectional area of the TECAR active electrode and US probe were similar, the actual tissue area between both TECAR electrodes (with 17×23 cm inactive electrode size) was greater than that of the US. So, TECAR appears to give more energy to the hamstring muscles [31].

In addition, although US therapy with a frequency of 1 MHz and an intensity of 2 W/cm2 may increase intramuscular temperature, the heat generated under the probe dissipated throughout the tissue because the probe was moving during the treatment time. Therefore, the US could be more efficient in heating smaller areas of the body [14][32]. Furthermore, we only had three treatment sessions, and US treatment may not have been sufficient to elicit as much effect as TT (moderate effect size vs. strong effect size, respectively). So, more research with a longer treatment time for larger muscles like hamstrings with more treatment sessions is required to clarify this assumption.

In our study, SS alone improved the flexibility of the hamstring more than the US combined with SS. Nuri et al. [33] showed that SS could increase ankle dorsiflexion ROM more than US therapy (24.18% vs. 4.54%). It seems that stretching alone can increase the extensibility of tissues [34] due to a decrease in the stiffness of the muscle-tendon unit [35][36]. Although it was assumed that the combined effects of US and SS could improve flexibility more than SS alone, this was not the case. It could be due to the large surface area of the treatment, which prevents the local tissue temperature from rising. In addition, in our study, SS was performed after US therapy, additional benefits of US therapy may be seen when used concurrently with US therapy. Similar to our results, Mohammadi et al. did not find a significant difference between TT plus SS and SS alone in terms of improving hamstring flexibility [24].

On the other hand, Kim et al. showed that 15 minutes of TT alone could immediately improve hamstring flexibility [23]. It has been reported that deep heating modalities like the US or short-wave diathermy combined with stretching could have more immediate effects on muscle flexibility than stretching alone [37]. Therefore, it is possible that if we had performed SS simultaneously with either of the two thermal modalities, we could find a greater improvement than SS alone.

The effect size of the AKE ROM was more than PKE in both the TT and SS groups, which could be due to the difference in neurophysiological mechanisms of the two tests [27]. Also, quadriceps muscle strength should be considered, which was not taken into account in our study [6][38]. The change in AKE ROM was almost 7 degrees (6.38°) after three treatment sessions of TT.Concerning the minimal detectable difference in AKE in subjects with a flexibility deficit (7°-8°) [39], it appears that three sessions of TT may be useful for clinical purposes. The SR test between the groups showed no statistical difference. This test has shown high test-retest reliability [40]; however, its results could not only be influenced by the hamstring length but also by factors including the flexibility of the extensor muscles of the spine [41]. As a result, the assessment of the hamstring length may be influenced by the degree of spinal flexion.

To the best of our knowledge, this study was the first single-blind randomized controlled trial comparing the effects of TT and US therapy on the flexibility of hamstring muscles. The present study explored the immediate and short-term effects of the two thermal modalities, providing insight into their potential benefits. In addition, the knee extension ROM tests and the SR test were used concurrently. In this way, it is thought that the flexibility of the hamstring muscle from both ends (proximal and distal attachments) can be assessed. In the present study, the long-term effects of TT and US therapy were not evaluated. Further studies with more treatment sessions and simultaneous application of TECAR or US combined with SS are suggested.

## Conclusion

This study showed that three sessions of TT plus SS could improve the length of hamstring muscles more than US therapy combined with SS in male athletes with hamstring shortness. However, based on the SR test the flexibility of hamstrings between the three groups was similar at the end of the interventions.

## Acknowledgments

We would like to thank the Research Deputy at the Tehran University of Medical Sciences. We are also grateful to the athletes who participated in this research. This research did not receive any specific grants from funding agencies in the public, commercial, or not-for-profit sectors.

## Conflict of Interest

The authors declare no conflict of interest.
